# CORRELATION BETWEEN PRE AND POSTOPERATIVE LEVELS OF GLP-1/GLP-2 AND WEIGHT LOSS AFTER ROUX-EN-Y GASTRIC BYPASS: A PROSPECTIVE STUDY

**DOI:** 10.1590/0102-6720201600040010

**Published:** 2016

**Authors:** Everton CAZZO, Martinho Antonio GESTIC, Murillo Pimentel UTRINI, José Carlos PAREJA, Elinton Adami CHAIM, Bruno GELONEZE, Maria Rita Lazzarini BARRETO, Daniéla Oliveira MAGRO

**Affiliations:** 1 Department of Surgery; 2 Research Laboratory in Metabology and Diabetes (Limed), State University of Campinas - Unicamp, Campinas, SP, Brazil

**Keywords:** Bariatric surgery, Obesity, Gastric bypass, Glucagon-like peptide 2, Glucagon-Like Peptide 1

## Abstract

**Background:**

The role of gut hormones in glucose homeostasis and weight loss achievement and maintenance after bariatric surgery appears to be a key point in the understanding of the beneficial effects observed following these procedures.

**Aim:**

To determine whether there is a correlation between the pre and postoperative levels of both GLP-1 and GLP-2 and the excess weight loss after Roux-en-Y gastric bypass (RYGB).

**Methods:**

An exploratory prospective study which enrolled 11 individuals who underwent RYGB and were followed-up for 12 months. GLP-1 and GLP-2 after standard meal tolerance test (MTT) were determined before and after surgery and then correlated with the percentage of excess loss (%EWL).

**Results:**

GLP-2 AUC presented a significant postoperative increase (945.3±449.1 vs.1787.9±602.7; p=0.0037); GLP-1 AUC presented a non-significant trend towards increase after RYGB (709.6±320.4 vs. 1026.5±714.3; p=0.3808). Mean %EWL was 66.7±12.2%. There was not any significant correlation between both the pre and postoperative GLP-1 AUCs and GLP-2 AUCs and the %EWL achieved after one year.

**Conclusion:**

There was no significant correlation between the pre and postoperative levels of the areas under the GLP-1 and GLP-2 curves with the percentage of weight loss reached after one year.

## RESUMO

**Racional:**

O papel de hormônios gastrointestinais sobre a homeostase glicêmica e a obtenção e manutenção da perda de peso após a cirurgia bariátrica parece ser elemento fundamental na compreensão dos benefícios observados após estes procedimentos.

**Objetivo:**

Determinar se há correlação entre os níveis pré e pós-operatórios de GLP-1 e GLP-2 com a perda do excesso de peso após o bypass gástrico em Y-de-Roux.

**Métodos:**

Estudo prospectivo exploratório que envolveu 11 indivíduos submetidos ao bypass gástrico, acompanhados por 12 meses. Os níveis GLP-1 e GLP-2 após um teste de refeição padrão foram determinados antes e 12 meses após a operação e então foram correlacionados com o percentual de perda do excesso de peso.

**Resultados:**

Houve aumento significativo da área sob a curva do GLP-2 após a operação (945,3±449,1 *vs.* 1787,9±602,7; p=0,0037); a área sob a curva do GLP-1 apresentou tendência não-significativa à elevação após o procedimento (709,6±320,4 *vs.* 1026,5±714,3; p=0,3808). O percentual médio de perda de peso foi 66,7±12,2%.

**Conclusão:**

Não houve nenhuma correlação significativa entre os níveis pré e pós-operatórios das áreas sob as curvas de GLP-1 e GLP-2 com o percentual de perda de peso atingido após um ano.

## INTRODUCTION

The role of gut hormones in glucose homeostasis and weight loss achievement and maintenance after bariatric surgery appears to be a key point in the understanding of the beneficial effects observed following these procedures. The significant weight loss following Roux-en-Y gastric bypass (RYGB) has been extensively reported^2^. At first, it has been regarded as an effect of the diminishment in the volumetric capacity of the stomach, caused by the creation of a 20-40 ml pouch, along with the malabsorption caused by the exclusion of about 250 cm of the small bowel from the food transit^9^ ^,^ ^7^ ^,^ ^18^. Nonetheless, more recently, several gastrointestinal hormones whose release is affected by the surgical anatomical changes were also enrolled in this process^15^.

The production and release of both glucagon-like peptide 1 (GLP-1) and glucagon-like peptide 2 (GLP-2) dramatically alter after surgery, especially when the procedures include duodenal exclusion or passage of more nutrients by the distal small bowel^13^ ^,^ ^20^ ^,^ ^21^. GLP-1 presents significant insulin-secretion and insulin sensitivity promoting effects, and appetite-regulating activities, whereas GLP-2 plays a more enterotrophic role related with optimizing the gut cell proliferation and nutrient absorption^10^ ^,^ ^13^. 

This study aimed to determine whether there is a significant correlation between the pre and postoperative levels of both GLP-1 and GLP-2 and the excess weight loss observed after RYGB.

## METHOD

The study has undergone evaluation and was approved by the local Research Ethics Board. Surgery was indicated based on the National Institutes of Health Consensus Statement criteria.

This is an exploratory prospective cohort study which enrolled 11 individuals with morbid obesity aged 18-65 years old which underwent RYGB from January 2011 through December 2012. Exclusion criteria were: smokers, carrier of chronic illnesses which could affect food intake and/or cause weight loss (cancer, liver failure, renal failure, AIDS), endocrine disorders (Cushing's disease, types 1 and 2 diabetes mellitus, Addison's disease), users of dipeptidyl peptidase-4 (DPP-IV) inhibitors, and users of drugs that could affect food intake and/or cause weight loss. Individuals were evaluated immediately before and 12 months after surgery.

All procedures were performed by the same surgical team and with the same technique. The main features of the RYGB were a 30 ml gastric pouch, a 100 cm biliopancreatic limb, a 150 cm alimentary limb, and a common limb consisting of the remainder of the small intestine.

Laboratory studies included the pre and postprandial curves of GLP-1 and GLP-2, following a standard meal tolerance test (MTT). GLP-1 and GLP- 2 levels were determined by means of an enzyme-linked immunosorbent assay (Elisa), and were performed serial dosages through a standard meal tolerance test (MTT) before and after surgery. After an overnight fast (12 h), subjects were submitted to standard MTT, based on a mixed meal containing 515 kcal (41.8% fat, 40.7% carbohydrates, and 17.5% protein). Blood samples were drawn for GLP-1 and GLP-2 at -15, 0, 30, 45, 60, 90, 120, 150, and 180 min. For GLP-1 and GLP-2 analysis, blood samples were collected in tubes with EDTA3 plus Sigma diprotin. Serum samples were stored in a freezer at -80°C for posterior analysis of GLP-1 and GLP-2 with specific Elisa kits (Elisa, Millipore - Billerica M.A). The following variables were also analyzed: age, gender, body mass index (BMI), weight, weight loss (WL), and percentage of excess weight loss (%EWL).

### Statistical analysis

The results were expressed as means±standard deviation (mean±SD). The area under the curve (AUC) of GLP-1 and GLP-2 was calculated by the trapezoidal rule. For the comparison of continuous measures before and after surgery, the ANOVA analysis was used. The pre and postoperative AUCs of GLP-1 and GLP-2 in the study group were correlated with the %EWL by means of the Spearman correlation tests. The significance level adopted was 5% (p<0.05). The software SSPS v.16.0 (Chicaco, IL, USA) was used for the analysis.

## RESULTS

Of 11 individuals who underwent RYGB and were followed-up for 12 months, 54.5% were female. At baseline, mean age was 36.7±8.2 years old; mean preoperative weight was 123.5±13.1 kg; mean preoperative BMI was 46.3±3.1 kg/m^2^. Overall morbidity was 9.1% and there was no mortality. Surgery led to significant decreases in weight (123.5±13.1 vs. 85.3±16.1 kg; p<0.001); BMI (46.3±3.1 vs. 32.1±6.0 kg/m^2^; p<0.001); mean weight loss was 38.2±15.2 kg; mean %EWL was 66.7±12.2%. Table 1 summarizes these findings.

**TABLE 1:** Characteristics of the individuals at baseline and 12 months after surgery

Age (years)	36.7±8.2		
Gender	Female: 54.5% Male: 45.5%		
%EWL	66.7±12.2%		
	Baseline	Postoperative	Value of P
Weight (kg)	123.5±13.1	85.3±16.1	<0.0001
BMI (kg/m^2^)	46.3±3.1	32.1±6.0	<0.0001

%EWL=percentage of excess weight loss; BMI=body mass index

After surgery, in the curve following MTT, there was a significant increase of GLP-1 levels only at 45 min. The GLP-1 AUC presented a non-significant postoperative increase (709.6±320.4 vs. 1026.5±714.3; p=0.3808). The GLP-1 IAUC also presented a non-significant increase following surgery (79.4±108.3 vs. 438.2±889.0; p=0.1414). Postoperatively, following MTT, significant increases in the curve of GLP-2 levels were observed in all times evaluated from 15 through 180 min. The GLP-2 AUC significantly increased after surgery (945.3±449.1 vs.1787.9±602.7; p=0.0037). The GLP-2 IAUC also presented a significant increase following surgery (44.0±306.1 vs. 947.5±604.0; p=0.0003). Table 2 shows the detailed findings of GLP-1 and GLP-2 levels at all the time points before and 12 months after surgery.

**TABLE 2:**
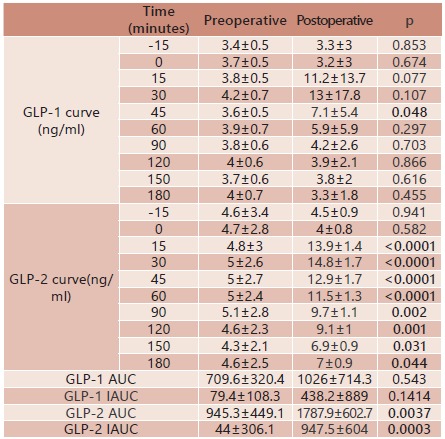
GLP-1 and GLP-2 levels and GLP-1 and GLP-2 AUCs/IAUCs following standard MTT before and 12 months after surgery

GLP-1=glucagon-like peptide 1; GLP-2=glucagon-like peptide 2; AUC=area under the curve; IAUC=incremental area under the curve; MTT=meal tolerance test

In regards of the correlation of the %EWL achieved after one year of surgery, there was not any significant correlation with both the pre and postoperative GLP-1 AUCs and GLP-2 AUCs. The complete correlation ranks are shown in Table 3.

**TABLE 3:** Correlation ranks between %EWL and GLP-1 and GLP-2 AUCs

	Correlation coefficient	p
%EWL versus Preoperative GLP-1 AUC	0.173	0.612
%EWL versus Postoperative GLP-1 AUC	-0.443	0.172
%EWL versus Preoperative GLP-2 AUC	0.500	0.117
%EWL versus Postoperative GLP-2 AUC	0.073	0.831

%EWL=percentage of excess weight loss; GLP-1=glucagon-like peptide 1; GLP-2=glucagon-like peptide 2; AUC=area under the curve

## DISCUSSION

The role of gastrointestinal hormones after bariatric surgery is an ever growing field of research. Several postsurgical changes have been reported and their specific roles in the glucose homeostasis, weight loss, food intake, and satiety are object of some controversy^20^ ^,^ ^21^.

A significant increase in the GLP-2 levels and a non-significant towards the increase of GLP-1 levels were observed following RYGB, comparable to previous reports in the literature.[8] These findings are probably linked to the structural changes in food transit caused by the procedure, mainly the passage of more nutrients by the distal small bowel and the duodenal exclusion^10^ ^,^ ^13^ ^,^ ^15^ ^,^ ^17^.

In the present study, significant correlations between the GLP-1 and GLP-2 levels and the weight loss achieved after RYGB were not found, signaling that it is possible that other factors should play a more relevant role in regards of weight loss and maintenance. Santo et al.^16^, studying late weight regain following RYGB, observed significantly lower postprandial levels of GLP-1 and glucose-dependent insulinotropic peptide (GIP) in individuals without sustained weight loss. DeHollanda et al.^6^, comparing individuals with postsurgical failed weight loss with those who achieved sustained weight loss 24 months after RYGB, observed lower increase in the postprandial GLP-1 and lesser suppression of ghrelin in individuals with failed weight loss, along with no differences in regards of GLP-2 and peptide tyrosine-tyrosine (PYY). Since weight regain and primary surgical weight loss failure are different phenomena, these previous findings are also difficult to be compared. Vidal et al.^20^, in a review study, supported the idea that the available data does not permit to conclude that GLP-1 is the major cause for the sustained weight loss achieved following surgery. In fact, there is a complex interplay of different gut hormones after surgery, along with the anatomical restrictive component of some techniques that are directly related with weight loss and maintenance after surgery. Furthermore, there are behavioral, social, and psychological factors that play significant roles in this regards as well^3^ ^,^ ^4^ ^,^ ^5^ ^,^ ^12^ ^,^ ^19^ ^,^ ^22^. 

This study has some limitations that must be noted. The small sample of individuals may limit further extrapolation. Since there are many other hormones possibly related to food intake and weight loss whose releases are affected by the surgical procedure, it would be preferable that all of these previously enrolled hormones had been evaluated. Moreover, there is no control group, which does not permit comparison with healthy individuals. Since it is consensual that the optimal weight loss achieved after RYGB is present at 18 months, the evaluation at the 12^th^ month may also compromise the observations. However, the results presented contribute to gain insight in regards of the possible roles of GLP-1 and GLP-2 in weight loss and weight maintenance.

Based on the findings of this study, the preoperative levels of GLP-1 and GLP-2 are not reliable predictors of the postoperative weight loss, as the postoperative responses in the both hormones do not correlate with the weight loss achieved as well. Weight loss and maintenance appear to be linked to a wider array of different gut hormones and physiologic responses affected by the surgical procedure, along the environmental factors related to the eating behavior of each individual^1^ ^,^ ^8^ Further research, especially in prospective controlled settings, is necessary to confirm and expand these findings.

## CONCLUSION

Pre and postoperative levels of GLP-1 and GLP-2 were not significantly correlated with the %EWL following RYGB within this sample.
